# Electricity inaccessibility across historically redlined and present-day disadvantaged areas in New York City

**DOI:** 10.1038/s41370-025-00767-1

**Published:** 2025-04-22

**Authors:** Alexander J. Northrop, Vivian Do, Perry E. Sheffield, Diana Hernández, Jane Clougherty, Joan A. Casey

**Affiliations:** 1 Department of Pediatrics, The Children’s Hospital of Philadelphia, Philadelphia, PA USA; 2 Department of Environmental Medicine, Icahn School of Medicine at Mount Sinai, New York, NY USA; 3 Department of Environmental Health Sciences, Columbia Mailman School of Public Health, New York, NY USA; 4 Department of Pediatrics, Icahn School of Medicine at Mount Sinai, New York, NY USA; 5 Department of Sociomedical Sciences, Columbia Mailman School of Public Health, New York, NY USA; 6 Department of Environmental and Occupational Health, Dornsife School of Public Health, Drexel University, Philadelphia, PA USA; 7 Department of Environmental and Occupational Health Sciences, University of Washington School of Public Health, Seattle, WA USA; 8 Department of Epidemiology, University of Washington School of Public Health, Seattle, WA USA

**Keywords:** Environmental Justice, Geospatial Analyses, Healthy Buildings, Climate Change

## Abstract

**Background:**

Electricity is crucial in sustaining livelihoods from turning the lights on at night, keeping the refrigerator running to avoid food spoilage, and powering electricity-dependent durable medical equipment such as nebulizers. Thus, electricity inaccessibility may result in adverse outcomes. Like other environmental burdens, electricity inaccessibility may be socially patterned, with disproportionate occurrence in racially and economically marginalized communities.

**Objective:**

To evaluate the 2017-2019 distribution of electricity inaccessibility – defined as power outages and energy insecurity – across historical and present-day measures of community racial disadvantage in New York City (NYC).

**Methods:**

We measured power outages with NYC 311 outage call reports and the System Average Interruption Frequency Index (SAIFI). We calculated energy insecurity as monthly average energy use, leveraging data from the New York State Energy Research and Development Authority. These three electricity inaccessibility metrics were estimated within both historical Home Owners’ Loan Corporation (HOLC) graded areas (A, ‘best’ through D, ‘redlined’) and present-day racial and economic Index of Concentrations at the Extremes (ICE) census tracts quartiles.

**Results:**

Our study covered 396 HOLC areas and 2218 census tracts in NYC. Historically A-graded areas had fewer 311 outage calls and lower SAIFI. Additionally, the rate of 311 outage calls in the present-day most disadvantaged census tracts was nearly six times that of the most privileged tracts. Persistently disadvantaged areas (i.e., both poor HOLC grade and high ICE) had more power outages than consistently advantaged areas. However, the present-day most disadvantaged census tracts still had more power outages than persistently disadvantaged areas.

**Impact:**

- This 2017–2019 New York City (NYC) study evaluated the distribution of three electricity inaccessibility metrics in relation to community privilege and disadvantage. Uniquely, we assessed the distribution across historically redlined areas and present-day census tracts. We defined electricity inaccessibility as power outages (311 calls and power interruptions) and energy insecurity (residential energy use). We found that 311 calls and power interruptions were more common in historically redlined areas, present-day disadvantaged census tracts, and persistently disadvantaged areas. These findings indicate proxies for historical racial discrimination, such as redlining, and modern-day community disadvantages impact the access to reliable electricity in NYC.

## Introduction

Electricity inaccessibility may take several forms, such as a lack of reliable power supply or an inability to afford sufficient energy to meet needs [1]. Power outages, a manifestation of unreliable access to electricity, can reflect system-related problems in the electrical grid or residence. For example, aging equipment in the electrical grid (e.g., transformer failure) or home (e.g., faulty wiring or switches) can expose individuals to power outages. Power outages can affect an entire area (e.g., neighborhood) or individual buildings (e.g., homes). Separately, energy insecurity, defined as “an inability to adequately meet basic household energy needs,” [1] reflects limited energy use due to economic, behavioral, and physical circumstances. For example, physical deficiencies in the home, combined with economic hardships, can drive people to adopt energy-limiting behavior [2], a form of energy insecurity. All forms of electricity inaccessibility may harm well-being, given the role of electricity in most functions of daily life [3].

Periods of electricity inaccessibility have consequences for online connectivity, powering residences, and the use of life-sustaining medical equipment, such as nebulizers and ventilators. A growing literature, mostly in high-income countries, finds that power outages can trigger adverse health outcomes, including carbon monoxide poisoning [3–5], chronic obstructive pulmonary disease exacerbations [6, 7], pregnancy complications [8], and all-cause mortality [9, 10]. Additionally, energy insecurity, which primarily impacts lower-income households, has been linked to worse mental health, poorer sleep quality, and cardiovascular conditions [11, 12]. Identifying inequities in energy accessibility becomes more critical with accelerating climate change. Climate change causes more frequent and severe weather, which results in more outages and more energy demand as households increase air conditioning (A/C) and heat use in response to extreme temperatures. This can add an additional economic burden to communities already facing energy insecurity.

Those facing energy insecurity make tradeoffs (i.e., “heat or eat”) and vigilant conservation [13, 14], and already experience other burdens such as poor health and material and financial hardship [1, 15]. For example, low-income families facing energy insecurity may insufficiently cool their homes during the summer [2, 16]. Low-income families also tend to live in lower-quality housing, which may have poor insulation, compounding energy insecurity issues [17, 18]. Thus, families may experience accentuated heat and cold indoors because of financial hardship, exposing them to health risks associated with temperature extremes [12].

As with housing and health, electricity inaccessibility may have been shaped by historically discriminatory practices and processes that continue to have reverberating present-day effects [19]. The Home Owners’ Loan Corporation (HOLC) appraised investment risk in racially discriminatory patterns in the 1930s, grading areas on an A to D scale, where grade D areas were “redlined,” representing high investment risk and no mortgage loan provision. These grades reflect racial discrimination in the housing market, which led to cyclical disinvestment in communities of color in the United States. They also provide a lens to characterize disadvantaged communities and the legacies of structural racism. Prior work found that lower-graded areas experience higher modern-day environmental exposures such as air pollution [20], land surface temperature [21], power plant siting [22], and less green space [23, 24] as well as worse health [19]. Thus, it is possible that such historical processes can contribute to differential modern-day exposures to electricity inaccessibility. Because HOLC originally incorporated housing quality into grade decisions, redlining practices were likely correlated with lower baseline housing quality and electrical infrastructure, as well as subsequent maintenance. Prior research has found associations between historical redlining and modern-day housing types [25]. Persistent housing characteristics (e.g., poor insulation) may continue to affect energy insecurity among residents today [26]. Historical disinvestment in energy infrastructure upkeep may also increase modern-day power outages in historically redlined areas as the energy grid ages, becoming susceptible to damage and requiring repair and reinvestment.

Electricity inaccessibility likely also varies across levels of present-day community disadvantage, as measured by indices such as the Index of Concentration at the Extremes (ICE). The racial and economic ICE measures “spatial social polarization,” identifying areas of concentrated disadvantage and privilege [27]. Present-day power outages are more common in socially vulnerable [28] and racially marginalized communities [29]. Concentrated disadvantage means community members also may face more energy insecurity, but to our knowledge, no studies have quantitatively assessed several components of electrical inaccessibility using a metric measuring both racial and economic disadvantage.

Our study objective was to examine the distribution of current electricity inaccessibility metrics across historically redlined communities based on HOLC grades and present-day racial and economic disadvantage at the census tract level in New York City (NYC). For 2017-2019, we measured electricity inaccessibility as outage-related 311 calls, neighborhood power outages, and electrical energy use and described their distribution by HOLC grade and ICE quartile. Each metric is intended to reflect different dimensions of electricity inaccessibility. 311 calls represent household-level power outages. Neighborhood power outages represent system-wide interruptions. Electrical energy use can signal energy insecurity when customers use less energy than required for optimal well-being. These data allowed us to assess whether 1) historical processes, 2) current disadvantage, or 3) both were associated with present-day electrical inaccessibility metrics in NYC.

## Methods

### Study design and spatial boundaries

We conducted a cross-sectional study assessing the distribution of electricity inaccessibility metrics (311 calls, power interruptions, and energy use) from 2017 to 2019 across historically redlined areas and present-day census tracts of varying levels of disadvantage in NYC.

We delineated communities of different HOLC grades using NYC HOLC boundaries and grades from the University of Richmond Inequality Mapping Project [30]. These digitized and georeferenced shapefiles contain the information from the original HOLC security maps, where areas were shaded to indicate mortgage lending risk. A-graded areas were considered to represent the lowest investment risk (‘best’ or most privileged), followed by B-, C-, and D-graded (least-privileged, i.e., “redlined”) areas [31, 32].

### Index of Concentration at the Extremes (ICE)

To characterize present-day disadvantage, we computed the racial and economic Index of Concentration at the Extremes (ICE) [27] for census tracts using data from the 2016-2020 American Community Survey [33]. Briefly, ICE measures racial and economic disadvantage by comparing the proportion of privileged and non-privileged groups and ranges from 0 (lowest concentration of households with racial and economic privilege) to 1 (greatest concentration of racial and economic privilege). We considered privileged groups to be white households making more than $99,999, which approximates the NYC 80^th^ percentile median household income from 2016-2020, and non-privileged groups to be non-white households making less than $44,999, which approximates the NYC 20^th^ percentile median household income in the same period. For our analyses, we categorized ICE into quartiles such that Q1 represented the greatest concentration of disadvantage and Q4 represented the greatest concentration of privilege. While customers and accounts were included in their respective datasets, we employed areal-weighted interpolation, using spatial weighting to determine the allocation of customers to HOLC boundaries using 2016-2020 American Community Survey data and the *areal* package [33, 34]. We leveraged the 2020 census tract boundaries [33].

### Power outages

We used a self-report measure (311 calls) and an objective measure (New York State Department of Public Service [NYS DPS]) to compare two different power outage metrics.

### 311 calls

Calls to 311, where city residents contact the City of New York for a variety of service requests [35], include calls regarding power outages. 311 calls indicate building-level power outages that occur at individual residences. We downloaded publicly available daily 311 call data from the NYC 311 agency. We included all 311 calls about electrical power outages from 2017-2019. These data included latitude, longitude, time, and date of the call. Based on the call location, we summed the total of calls within each historical HOLC and 2020 census tract boundary and calculated the yearly household rate (per 1000 households) of outage-related 311 calls.

### SAIFI

Power outages can be measured in several ways [28, 36]. The NYS DPS provided data on electrical customers within power operating localities (POL). A POL is the smallest geographic unit within which the NYS DPS reports power outage data. The area of a POL approximates the size of Zip Code Tabulation Areas (ZCTAs), and the median number of customers served within a POL is 1,314. An electrical “customer” is an electricity end-user, including residential, commercial, and industrial electrical meters. For example, a home and a business would each be a customer and are indistinguishable from each other in this dataset. For each POL, we had information on the total number of customers served and customers without power every 30 minutes from 2017-2019. We aggregated this information into hourly increments for data harmonization. Only POLs with ≥95% of possible hours were included in the analyses. We employed the MICE package [37] in R to conduct imputation using five iterations for any missing data.

We used a proxy for the System Average Interruption Frequency Index (SAIFI) [38]. Unlike 311 calls that reflect household-level outages, SAIFI is an established metric for electrical system reliability and measures the frequency of non-momentary electric outages per customer, which is operationalized as any outage lasting five minutes or longer [38]. We calculated a modified SAIFI covering our three-year study period as the total number of customer-hours without power from 2017-2019 divided by the yearly average number of customers served within a HOLC boundary (**Equation 1**). For example, a POL with 2500 customer-outage-hours and 10,000 customer-years from 2017-2019 would have a three-year SAIFI of 0.25. An electrical customer can be residential or a business customer, so either of these customer types can contribute to the customer-outage hours, customer-years, and by extension SAIFI metric. A higher SAIFI indicates less system reliability, while a lower SAIFI indicates greater system reliability. Electrical customers faced a yearly SAIFI of 1.35 from 2017-2019 in the United States [39]. We then performed areal-weighted interpolation from POLs to HOLC boundaries and census tracts as described previously [30].

#### Equation 1. Modified SAIFI

X represents the number of customers-outage-hours per POL, p, over the study period. C_y_ represents the number of average customers per year, y. ${{{\rm{\delta }}}}$ represents the number of years. This equation was adapted from the Institute of Electrical and Electronics Engineers. 

$${{SAIFI}}_{{average}}=\frac{{X}_{p}}{{C}_{{py}}\delta }$$

### Energy use

Electrical energy use per residential customer can serve as a proxy for energy insecurity when customers use less energy than required for optimal well-being. Unlike 311 calls and SAIFI, which represent power outages, residential energy use may reflect energy use behaviors in response to the building and economic circumstances. Higher residential energy use rates may suggest less energy insecurity, while lower residential energy use rates may suggest more [15]. We downloaded publicly available energy use data from 2017-2019 from the New York State Utility Energy Registry (UER), which collects these data directly from utilities. We filtered these UER data to residences, excluding businesses. Data were provided monthly for NYC ZIP codes for residences, which we then linked to ZCTAs using the Uniform Data System Mapper crosswalk [40]. We calculated residential customers’ average monthly megajoules (MJ) of electrical energy use by ZCTA. For the 2% of missing months of residential energy use data, we employed the *MICE* package [37] in R to conduct imputation using one-hundred iterations. We used areal-weighted interpolation from ZCTAs to HOLC boundaries and census tracts to estimate community residential energy use each month, as described before.

### Correlation of Metrics

Before characterizing the distribution of electricity inaccessibility metrics, we assessed the correlation between metrics (i.e., 311 outages, residential energy use, and SAIFI) to examine whether they captured overlapping dimensions of electricity inaccessibility. We used Spearman’s rank correlation coefficients to evaluate each pair of electricity inaccessibility metrics across historical HOLC boundaries and separately present-day census tract boundaries. We found limited correlation and thus conducted analyses for each electricity inaccessibility metric separately (Supplementary Tables S1 and S2).

### Statistical analysis

Analyses included all NYC HOLC areas and census tracts with >30 households (2,217 (95.3%) of 2,327 total tracts), customers, or accounts, depending on the metric. This resulted in exclusions of 1 (0.3%) HOLC-graded areas and 109 (4.7%) of census tracts. We evaluated the distribution of each electricity inaccessibility metric, 311-reported outages, power interruptions (SAIFI), and energy use across historical HOLC grades and present-day census tract ICE quartiles. To assess the correlation of our three electricity inaccessibility metrics, we calculated Spearman’s rank correlation coefficient within historical HOLC and present-day census tract boundaries. We employed non-parametric Wilcoxon Signed-Rank tests [41] to conduct pairwise comparisons between HOLC grades (A versus D areas) and ICE quartiles (Q1 – highest concentration of disadvantage versus Q4 – highest concentration of privilege) with grade A areas and Q4 census tracts designated as the referent distribution.

### Secondary analyses

Power outages and energy insecurity may vary seasonally [28]. For a secondary analysis, we stratified 311 calls, SAIFI scores, and energy use by warm versus cool season. As done in a prior power outage study in NYC [42], we defined the “warm” season as May – September and the “cool” season as October – April. We then re-ran analyses by season.

Additionally, areas ungraded by the Home Owners’ Loan Corporation may have a unique relationship with electricity inaccessibility [43]. Thus, to assess this, we used an aggregated ungraded boundary that comprised all land areas not covered by a HOLC boundary but covered by the ICE census tract boundaries. Using the aforementioned areal interpolation techniques and direct allocation of 311 outage calls, we determined the 311 outage rate, SAIFI, and energy use per household in the HOLC-ungraded areas of New York City.

Areas can become disadvantaged or privileged over time, and it is possible that persistently disadvantaged areas have more electricity inaccessibility compared to consistently advantaged areas due to historical disinvestment. After interpolating electricity inaccessibility metrics to historical- and present-day boundaries, we rasterized both the HOLC boundaries and census tracts. We then converted these rasters to comparable 100 m by 100 m grids. All the transformed HOLC 0.01 km^2^ squares that overlapped with the ICE 0.01 km^2^ squares were included in this analysis.

## Results

### Historically redlined areas and electricity inaccessibility metrics

Our analysis of 396 historically HOLC-graded A-D areas with electrical inaccessibility data for 2017-2019 covered 84.8% of the present-day NYC households (Supplementary Fig. S1**)**. We included 386 (97.2%) areas in 311-call analyses, 396 (99.7%) in SAIFI analyses, and 379 (95.5%) in energy use analyses. Most of these areas received a C grade (n = 189 [47.7%]), followed by D grade (n = 119 [30.1%]), B grade (n = 72 [18.2%]) and A grade (n = 16 [4.0%]). HOLC grades varied by borough such that grade D areas were concentrated in Manhattan, the South Bronx, and parts of outer Brooklyn and Queens (Supplementary Fig. S2).

**Fig. 1:**
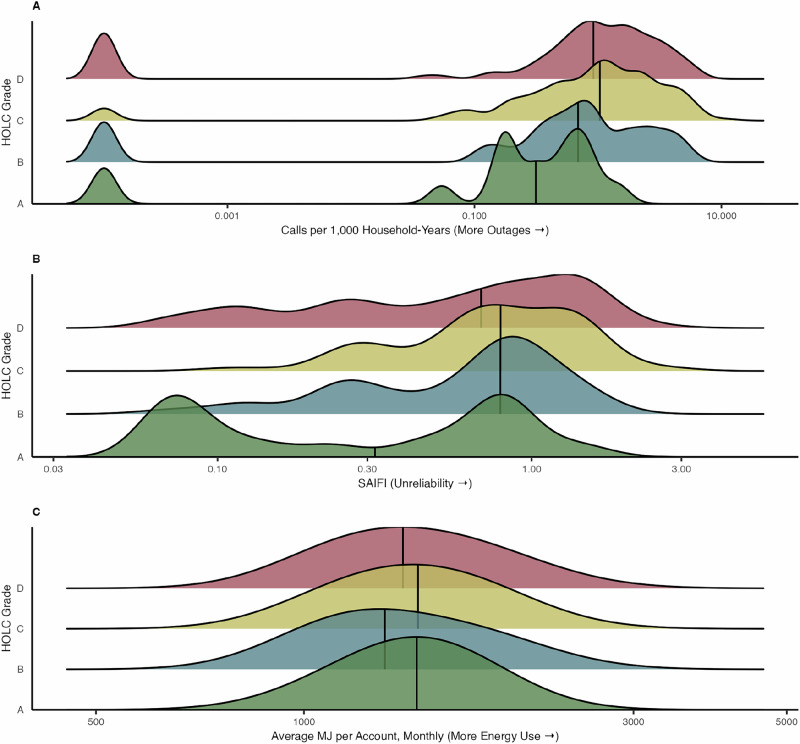
Ridgeline plot of the prevalence of electricity inaccessibility metrics in New York City, 2017-2019 by historical HOLC grades. **A** 311 outage calls per 1000 households, (**B**) system-related power outages, and (**C**) electrical energy use. Grade A (green) was “best,” grade B (blue) was “still desirable,” grade C (yellow) was “definitely declining,” and grade D (red) was “hazardous.”.

**Fig. 2:**
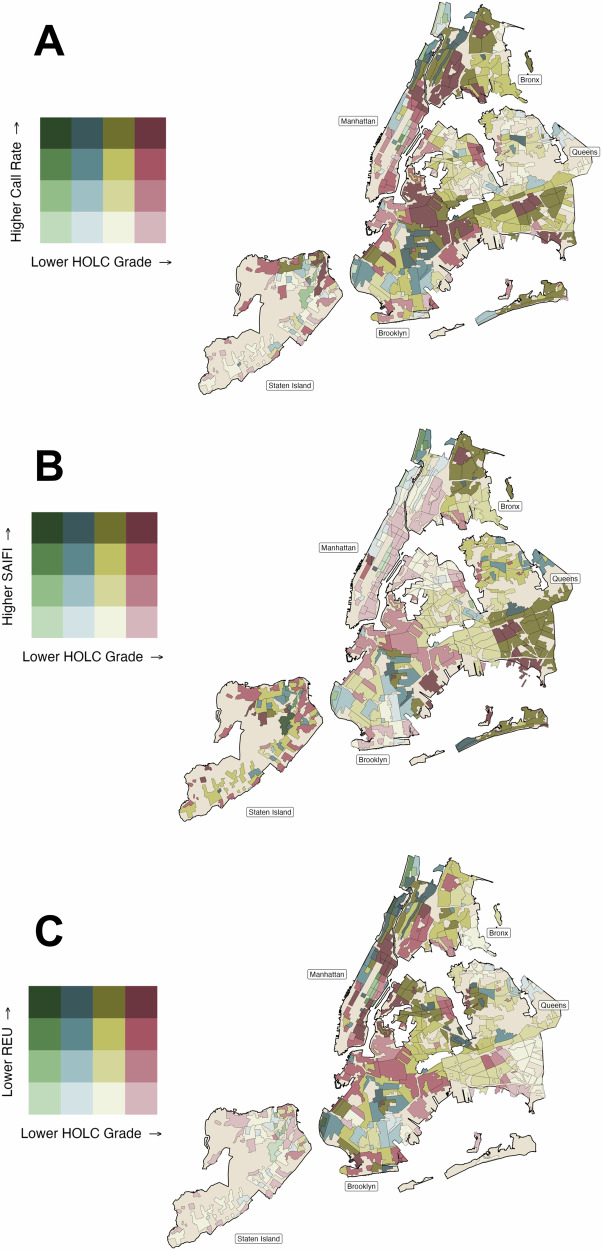
Spatial distribution of electricity inaccessibility metrics in New York City, 2017-2019 by historical HOLC grades. **A** 311 outage calls per 1,000 households, (**B**) system-related power outages, and (**C**) electrical energy use. Areas with a historical grade and the respective electricity inaccessibility metric are shown. Grade A (green) was “best,” grade B (blue) was “still desirable,” grade C (yellow) was “definitely declining,” and grade D (red) was “hazardous.”.

We observed different levels of cumulative outage-related 311 outage calls per 1,000 households by A- versus D-graded areas (p-value < 0.01, Fig. 1A). 311-reported outage rates were most common in C-graded areas (median rate of 1,000 household-years: 1.04; IQR: 0.39-2.27) and D-grades areas (median: 0.92; IQR: 0.37-1.93), where outage reports were three times more common than in A-graded communities (median: 0.31; IQR: 0.17-0.67) (Fig. 1A, Supplementary Table S3). C-graded and D-graded areas with high 311 outage calls per 1,000 households typically occurred in the Bronx, Northern Manhattan, Queens, and Brooklyn (Fig. 2A).

We also found differences in SAIFI by A- versus D-graded areas (p-value = 0.02). B-, C-, and D-graded areas experienced more electrical interruptions than A-graded areas. A-graded areas had a median of 0.33 (IQR: 0.08-0.80) yearly outages per customer, compared to a median of 0.69 (IQR: 0.26-1.34) yearly outages per customer in D-graded areas (Fig. 1B). D-graded areas with high SAIFI were in Queens, and the highest SAIFI metrics were concentrated in C- and D-graded areas in the outer Brooklyn region (Fig. 2B).

Median residential energy use per household did not differ statistically between A- versus D- graded HOLC boundaries (p-value = 0.93) (Fig. 1C). Instead, energy use appeared to pattern by borough rather than HOLC grades. Residences in Manhattan, Queens, the Bronx, and Brooklyn tended to have lower energy use than Staten Island (Fig. 2C).

In our seasonal secondary analysis, 311-reported outages and SAIFI results were consistent with the main analysis. D-graded areas had a greater number of 311-reported outages during the cool season (i.e., October - April) (median: 0.85 call rate, IQR: 0.14-1.66) versus A-graded areas (median: 0.31 call rate, IQR: 0.17-0.48). During the warm season (i.e., May - September), D-graded areas also faced more 311-reported outages (median: 0.95, IQR: 0.33-2.27) versus A-graded areas (median: 0.15, IQR: 0-0.65) (Supplementary Fig. S3). Power interruptions measured by SAIFI were more common in D-graded areas (median: 0.31, IQR: 0.13-0.40) compared to A-graded areas during the cool season (median: 0.20, IQR: 0.06-0.36) and warm season (SAIFI in D-graded area median: 0.35, IQR: 0.12-0.59 versus A-graded area median: 0.13, IQR: 0.02-0.23). In contrast, when considering residential energy use by season, we identified lower warm-season energy use in D-graded areas (median: 1557 average MJ per account, IQR: 1355-1890) compared to A-graded areas (median: 1734 average MJ per account, IQR: 1450-1888).

### Present-day ICE census tracts and electricity inaccessibility

Our present-day ICE analyses included 2,218 census tracts containing 3,191,608 households. We included 2,218 (95.3%) census tracts in 311 call analyses, 2,118 (95.3%) in SAIFI analyses, and 2,193 (94.2%) in energy use analyses (Supplementary Fig. S4). Q1 census tracts (most concentrated disadvantage) were located mostly in the Bronx and Brooklyn, while Q4 census tracts (most concentrated privilege) were concentrated in Staten Island and Manhattan (Supplementary Fig. S5).

**Fig. 3:**
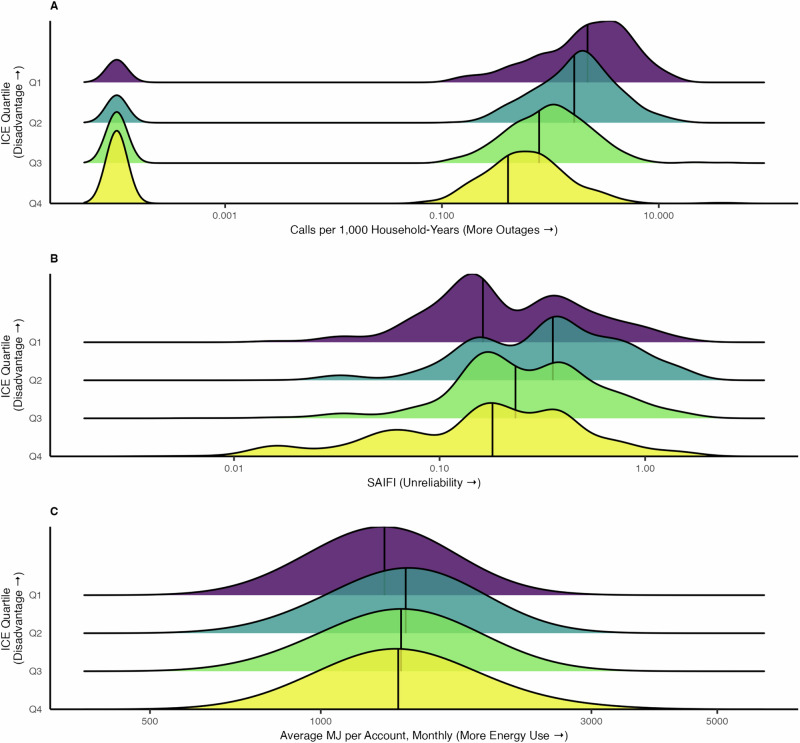
Ridgeline plot of the prevalence of electricity inaccessibility metrics in New York City, 2017-2019 by 2016-2020 census tract level quartiles of racial and economic Index of Concentration at the Extremes. **A** 311 outage calls per 1,000 households, (**B**) system-related power outages, and (**C**) electrical energy use. Q1 represents quartile with greatest concentration of modern-day disadvantage, and Q4 represents quartile with greatest concentration of modern-day privilege.

**Fig. 4:**
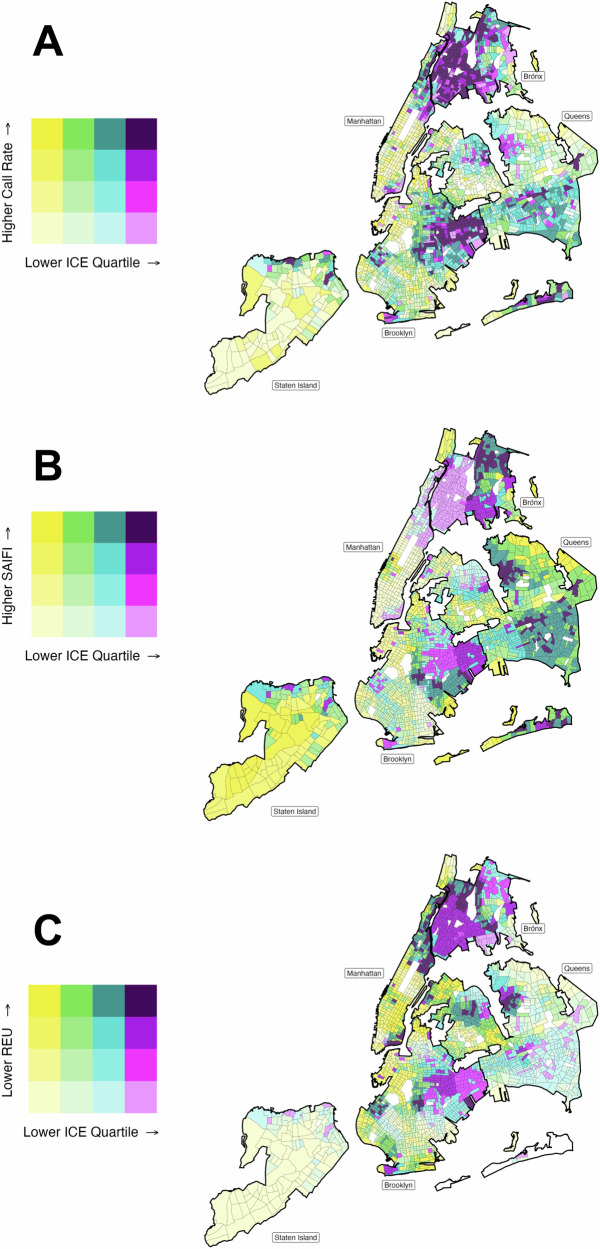
Spatial distribution of electricity inaccessibility metrics in New York City, 2017-2019 by 2016-2020 census tract level quartiles of racial and economic Index of Concentration at the Extremes. **A** 311 outage calls per 1,000 households, (**B**) system-related power outages, and (**C**) electrical energy use. Census tracts with >30 households were included.

Rates of 311 outage-related calls differed by Q1 (most disadvantaged) versus Q4 (most privileged) ICE quartiles (p-value < 0.01), with monotonically higher 311 outage calls per 1,000 households at higher levels of concentrated disadvantage. Q1 census tracts had the highest 311 outage calls per 1,000 households (median: 2.20, IQR: 0.83 - 4.01), over five times the Q4 call rate (median: 0.41, IQR: 0 - 0.84) (Fig. 3A**;** Supplementary Table S4). Census tracts with high concentrated disadvantage and high 311 outage calls per 1,000 households were in the outer Bronx and outer Queens (Fig. 4A, Supplementary Table S4).

SAIFI distribution slightly differed between Q4 (most privileged) and Q1 (most disadvantaged) census tract ICE quartiles (p-value = 0.02), though Q4 tracts (median: 0.43, IQR: 0.24-0.70) and Q1 tracts (median: 0.45, IQR: 0.25-0.76) experiencing fewer interruptions than Q2 and Q3. In fact, Q2 census tracts had the most interruptions (median: 0.69, IQR: 0.43-1.41) (Fig. 3B). The tracts with the most concentrated disadvantage and frequent electrical interruptions were in the outer Bronx and outer Queens (Fig. 4B).

Energy use in Q4 (most privileged) versus Q1 (most disadvantaged) ICE quartiles differed significantly (p-value < 0.01), with Q1 census tracts having the lowest median energy use throughout the year (median: 1234 monthly MJ per account, IQR: 1147-1358) (Fig. 3C). Areas with both low energy use and high concentrated disadvantage were in outer Brooklyn, the Bronx, and Northern Manhattan (Fig. 4C).

In our seasonal secondary analyses, the results were mostly consistent with the overall findings. During the cool season (i.e., October – November), 311 outage rates were higher among the most disadvantaged census tracts (median = 1.79, IQR: 0.59-3.60) compared to the most privileged tracts (median: 0.28, IQR: 0.00-0.75). During the warm season (i.e., May – September) differences between Q1 (most disadvantaged) and Q4 (most privileged) persisted (median = 2.14, IQR: 0.74-4.56 versus median = 0.34, IQR: 0.00-1.01, respectively) (Supplementary Fig. S6). Power interruptions measured by SAIFI were similar between Q1 (most disadvantaged) (median: 0.15, IQR: 0.12-0.38) and Q4 (most privileged) census tracts (median: 0.16, IQR: 0.08-0.23) during the warm season. However, SAIFI differed between Q1 (median: 0.30, IQR: 0.14-0.57) and Q4 census tracts (median: 0.23, IQR: 0.08-0.36) during the cool season. When considering residential energy use by season, residential households in Q1 census tracts persistently used less energy than households in Q4 census tracts.

In our ungraded HOLC secondary analysis, the 311 outage rate was 0.28 per 1000 household-years in the ungraded boundary, lower than HOLC grades A through D. SAIFI in the ungraded area was 0.90, a higher value than HOLC grades A through D. Finally, the energy use in ungraded HOLC areas was also lower than HOLC grades A through D at a monthly average of 1335 MJ/account.

### Relationship between persistent community disadvantage and present-day electricity inaccessibility

We also considered the potential cumulative implications of an area being persistently disadvantaged, measured by being graded D in the 1930s and Q1 (most disadvantaged) of ICE in 2016-2020. We had data on historical HOLC grades and ICE quartiles for 53.4% of the 783 km^2^ of land area in NYC. Broadly, we found that historically yellow-lined or redlined areas (HOLC grades C and D) tended to become census tracts with present-day concentrated disadvantages. While areas with historical HOLC grades A and B became census tracts with concentrated privilege (Supplementary Fig. S7). For example, 40.1% (4,965 0.01 km^2^ gridded square units) of grade D areas became Q1 (most disadvantaged) ICE census tracts, and another 25.3% became Q2 ICE census tracts, whereas only 0.2% (2 square areal units) of grade A areas became Q1 census tracts (Supplementary Table S5). Grade C areas had the most heterogeneity in their present-day ICE quartile corollaries, with 28.9% becoming Q1, 32.2% becoming Q2, 24.9% becoming Q3, and 14.0% becoming Q4 (most privileged). Notably, certain areas with a D-grade became more privileged today, such as the large portions of the Upper East Side that were D-graded but now fall in the Q4 ICE category.

**Fig. 5:**
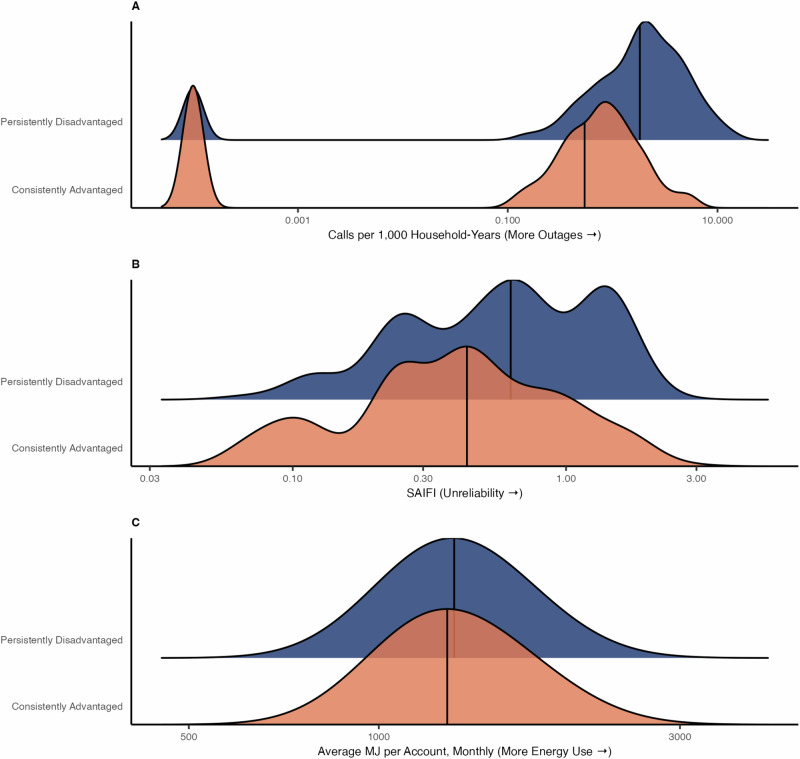
Ridgeline plot of the consistently advantaged and persistently disadvantaged areas in New York City and their 2017-2019 prevalence. **A** 311 outage calls per 1000 households, (**B**) system-related power outages, and (**C**) electrical energy use. Consistently advantaged refers to areas historically graded A and presently categorized as economically and racially privileged census tracts (Q4 ICE). Persistently disadvantaged refers to areas historically graded D and presently categorized as economically and racially disadvantaged census tracts (Q1 ICE).

We observed differences in 311 outage calls per 1,000 households and power interruptions when comparing persistently disadvantaged (C or D grade [historical disadvantage] + Q1 or Q2 [present-day disadvantage]) and consistently advantaged (A or B grade [historical privilege] + Q3 or Q4 [present-day privilege]) areas (Supplementary Table S6). Persistently disadvantaged areas had higher median 311 outage calls per 1,000 households versus consistently advantaged areas (1.82 outage calls per 1,000 households, IQR: 0.71-3.42 versus 0.54, IQR: 0.12-1.10; p-value < 0.01). However, the 311 outage rate in persistently disadvantaged areas did not exceed that of present-day Q1 tracts alone. Power interruptions were also more common in persistently disadvantaged versus consistently advantaged areas [average median SAIFI 0.63, (IQR: 0.28-1.34)] versus 0.43, (IQR: 0.24-0.80) (p-value < 0.01)] and the SAIFI in consistently advantaged areas was similar to that in HOLC A areas alone or present-day Q1 ICE tracts. We did not observe statistically significant differences in energy use (Fig. 5). Despite a relationship between redlining and ICE with measures of electricity inaccessibility, our results do not suggest a cumulative effect for areas of persistent disadvantage.

## Discussion

Our NYC study assessed the distribution of electricity inaccessibility metrics (i.e., 311 outage rates, power interruptions, and average residential energy use) from 2017-2019 across historically redlined areas and present-day concentrated disadvantage in census tracts. In our historical analyses, grade A areas experienced lower 311 outage calls per 1,000 households and fewer electrical interruptions from 2017-2019. In our present-day analyses, we observed the greatest disparity related to 311 outage calls per 1,000 households. Compared to the most privileged census tracts, the most disadvantaged ones faced nearly six times the rate of 311 outage calls. Communities identified as persistently disadvantaged, that is, those redlined in the 1930s and the most disadvantaged ICE quartile in 2020, had higher 311 outage calls per 1,000 households and power interruptions compared to their consistently advantaged counterparts. However, census tracts with the greatest present-day disadvantage still had higher 311 outage calls per 1,000 households and more power interruptions than the persistently disadvantaged areas.

Our results support prior studies that found outages disproportionately burden marginalized communities. Marcotullio et al. found that in NYC, 311 calls were more frequent in low-income areas and communities of color [44]. Other studies relying on utility data to measure power outage exposure observed that outages can be longer and more frequent in Hispanic [29], low-income [45], and socially vulnerable communities [28]. Studies thus far have investigated power outage disparities using metrics of present-day disadvantage. Our study contributes to the literature by finding that outages defined as 311 outage rates and SAIFI were more common in present-day disadvantaged areas and historically redlined areas of NYC. Disparities were more pronounced in present-day census tract ICE quartiles compared to HOLC redlined areas, suggesting that present-day markers may be better indicators for disparities of electricity inaccessibility compared to historical metrics due in part to gentrification. Future studies can investigate which dimensions of disadvantage (e.g., economic) are most associated with electricity inaccessibility to identify intervention points to reduce it.

Currently, few studies consider the role of long-term community disadvantage or advantage in relation to environmental exposures. Swope et al. outlined a theoretical framework demonstrating how historical redlining practices, along with other discriminatory policies such as racial zoning, could influence neighborhood-level characteristics and place-based risk factors [19]. The authors underscored the cyclical impact of historical policies on present-day environmental exposures. Our study found that persistently disadvantaged areas experienced more power outages than consistently advantaged ones. One reason could be long-term disparities in energy infrastructure investment, maintenance, and upgrades. However, the observed inequalities between persistently disadvantaged versus consistently advantaged areas were not larger than those for present-day comparisons alone. One possible reason for the inequality between the most privileged and disadvantaged geographic units could be driven by either historical or present-day measures of advantage alone, albeit not synergistically. For individual-reported 311 outage rates, our present-day marker of inequality (ICE) had a higher magnitude of inequality between the most privileged and disadvantaged areas compared to the historical measure (HOLC). In NYC, 311 outages were previously shown to spatially cluster in high-poverty neighborhoods [44]. We found that 311 outage rates were correlated with modern-day markers of disadvantage. In contrast, HOLC grades, rather than present-day disadvantage, had the greatest magnitude of inequality for SAIFI. Historical processes and decisions may have contributed to infrastructure investments and system-level power outages today. Accounting for spatial changes in advantage and disadvantage can be important in identifying areas facing a cycle of disadvantage. It is important to acknowledge that historically disadvantaged areas can become privileged areas and vice versa. For example, urban renewal and gentrification can redirect investments into neighborhoods, reversing the trend of disadvantage; however, these very processes can simultaneously displace and harm under-resourced individuals native to these areas [46, 47]. Future research should examine the distribution of electricity inaccessibility considering these processes including tracking key turning points.

We did not observe differences in energy use between persistently disadvantaged and consistently advantaged areas. This may be due to the competing influences of resource wealth and resource constraints. Higher-income residences may have improved insulation and higher-efficiency appliances, reducing the relative energy expenditure per residence despite few resource constraints [48]. Conversely, poorer households may limit energy expenditures despite housing with poorer insulation and fewer available energy-efficient appliances. In essence, these findings may indicate that behavioral and economic forces may counteract energy efficiency, resulting in a null finding. Moreover, higher-income households have more solar installed, effectively reducing their energy use from energy utility companies [49]. Thus, the energy use data may not capture the relative “value” of energy expenditures among persistently disadvantaged and consistently advantaged areas.

Surprisingly, we found no correlation between SAIFI and 311 outage calls per 1,000 households. This could be because each metric captures different dimensions of power outages. As noted, SAIFI measures average power interruptions per customer across a spatial area (e.g., census tract) and measures the overall performance and quality of the electrical grid. Not everyone in the spatial area will experience the outages that drive the SAIFI. On the other hand, residents must report via 311 calls when they experience a power outage. These outages could be due to system performance or more proximal causes related to housing, such as wiring issues or breaker issues. Additionally, the lack of correlation could be because SAIFI was calculated using residential and business customers, while 311 outage calls were for residential customers alone. Differences between these constructs may arise for other reasons, such as where individuals live who are more likely to dial 311.

Our study had several strengths. One strength is that we used historical HOLC boundaries and present-day ICE quartiles within census tracts. Considering both illuminates the extent to which historical and present-day disadvantage is correlated with electricity inaccessibility. Studies often focus on a single aspect of electricity inaccessibility, but we include three. Another strength is that the study compared 311 calls per 1,000 households and area-level SAIFI. Future research should recognize these differences when linking power outages to health outcomes, as each metric may have different health pathways. For example, building-level power outages prevent temperature-controlling devices (e.g., air conditioners, fans) from working, leading to prolonged exposure to extreme temperatures and, by extension, adverse health events [46, 47]. On the other hand, system-wide outages that affect large areas may also contribute to psychosocial stress due to unreliable power, concerns about criminal activity, and possibly health consequences [50, 51].

A study limitation is that electricity inaccessibility was measured at the area level instead of the individual level. Data on energy insecurity have previously been collected at the individual level [11, 12, 15], but we relied on energy use data reported by utilities originally at the ZIP code level. Additionally, energy use proxied for energy insecurity in our study because we lacked data on behaviors and financial constraints related to energy insecurity. For example, areas in Staten Island had high energy use, possibly driven by higher square-foot residences. Future studies should consider collecting individual-level data on energy insecurity. Our analyses were purely descriptive and did not explore factors that may explain the observed differences in energy insecurity metrics across historical- and present-day boundaries of privilege and disadvantage. A future direction could be identifying factors contributing to such differences through multivariate modeling. Though our study strengths include multiple spatial boundaries representing historical and present-day markers of disadvantage, we relied on areal-weighted interpolation to transform the data to the geometries of interest. This method could lead to different measurement error within our spatial boundaries and precludes direct comparison of the two. Thus, the correlation coefficients across metrics may differ based on geographic boundaries, as seen in Supplementary Table 1 and Supplementary Table 2. Finally, our SAIFI metric did not capture the proportion of residential, industrial, or commercial customers with or without power, which limits our ability to identify the classification of consumers’ electrical system reliability. Moreover, these data do not include customers who received a disconnection from the energy utility company.

Our study found disparities in electricity inaccessibility for historically redlined and present-day boundaries of disadvantage. Historical and present-day disadvantages can shape electricity inaccessibility, with potential repercussions for health and health equity. Energy-related policies should consider strategies to promote equitable investment in electrical infrastructure.

## Supplementary information

Supplementary Material

## Data Availability

311 outage calls data are publicly available at https://data.cityofnewyork.us/Social-Services/311-Service-Requests-from-2010-to-Present/erm2-nwe9. The power interruptions data is publicly available at https://dps.ny.gov/electric. Energy use data is publicly available at https://data.ny.gov/Energy-Environment/Utility-Energy-Registry-Monthly-Community-Energy-U/m3xm-q3dw/about_data. HOLC boundaries can be found at https://dsl.richmond.edu/panorama/redlining/data. Data used to create the Index of Concentration at the Extremes is from https://www.nhgis.org/. Code used to process and analyze data is accessible at https://github.com/ajnorthrop/redlining-electricity-inaccessibility.
