# Supplementary material for: Electricity inaccessibility across historically redlined and present-day disadvantaged areas in New York City
Source: J Expo Sci Environ Epidemiol. 2025 Apr 22;35(5):848–58. doi: 10.1038/s41370-025-00767-1 (PMC12401720; doi:10.1038/s41370-025-00767-1)
Supplement: Supplementary file 1 — Supplementary Material [file 41370_2025_767_MOESM1_ESM.docx]

**Supplementary Material**

| **Metric Comparison** | **Spearman Correlation Coefficient** | **p-value** |
| --- | --- | --- |
| **Residential energy use-outage rate (311 calls)** | -0.24 | <0.01 |
| **Residential energy use-SAIFI** | 0.56 | <0.01 |
| **Outage rate (311 calls) -SAIFI** | -0.07 | 0.15 |

**Supplementary Table S1**. Spearman’s rank correlation matrix of present-day electrical inaccessibility metrics at the historical HOLC grade area level.

| **Metric Comparison** | **Spearman Correlation Coefficient** | **p-value** |
| --- | --- | --- |
| **Residential energy use-outage rate (311 calls)** | -0.07 | <0.01 |
| **Residential energy use-SAIFI** | 0.41 | <0.01 |
| **Outage rate (311 calls) -SAIFI** | 0.08 | <0.01 |

**Supplementary Table S2**. Spearman’s rank correlation matrix of present-day electrical inaccessibility metrics at the 2020 census tract level.

**Supplementary Figure S1.** Flowchart of NYC HOLC areas included in the study after exclusion criteria.

**
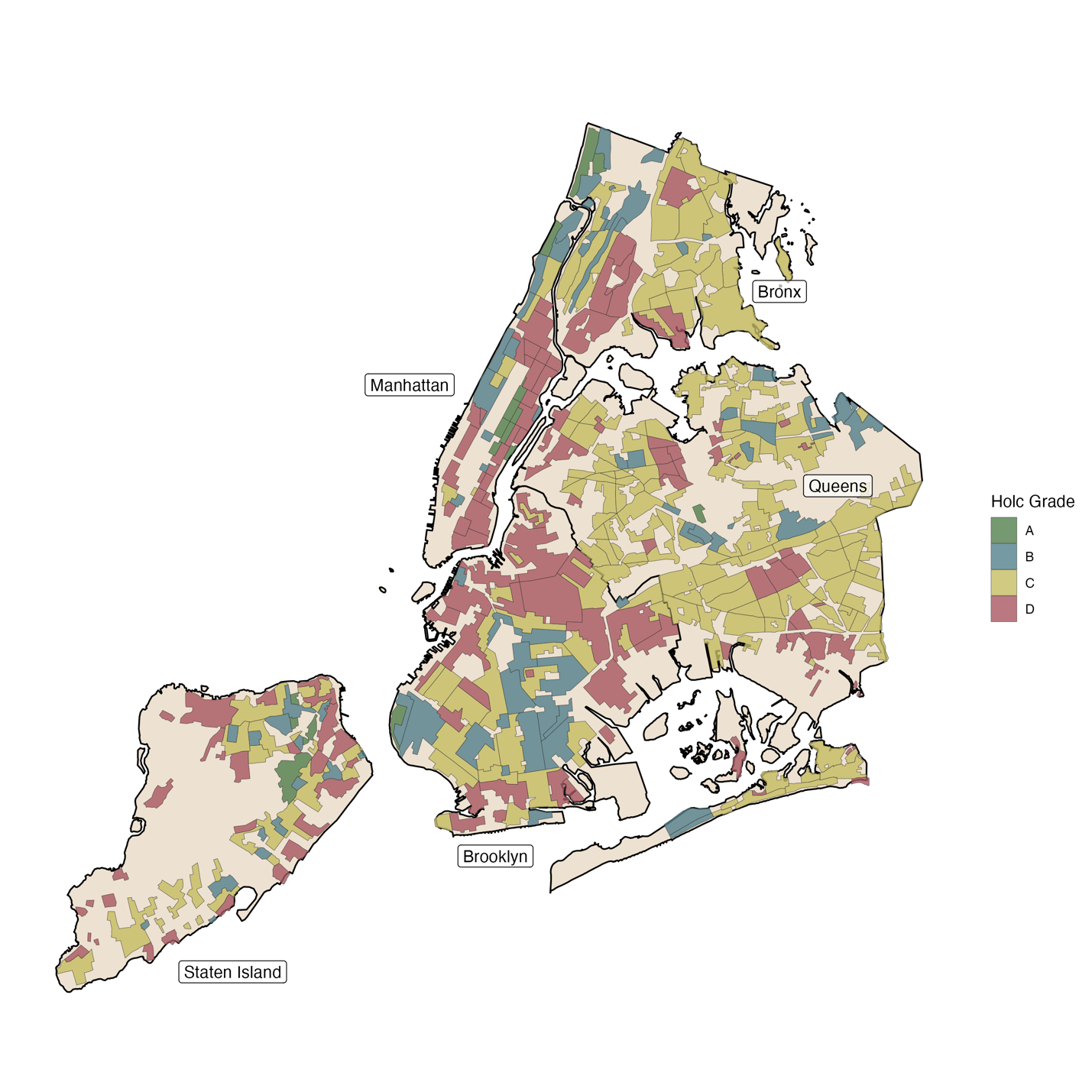
**

**Supplementary Figure S2.** NYC HOLC areas. Grade A (green) was “best,” grade B (blue) was “still desirable,” grade C (yellow) was “definitely declining,” and grade D (red) was “hazardous.”

|  | HOLC grade | Min. | Q1 | Median | Mean | Q3 | Max. | P-values |
| --- | --- | --- | --- | --- | --- | --- | --- | --- |
| 311 outage rates (calls/1000 customers) | D | 0 | 0.37 | 0.92 | 1.37 | 1.93 | 6.23 | <0.01 |
|  | C | 0 | 0.39 | 1.04 | 1.58 | 2.27 | 10.98 | <0.01 |
|  | B | 0 | 0.30 | 0.69 | 1.19 | 1.68 | 5.33 | 0.04 |
|  | A | 0 | 0.17 | 0.31 | 0.43 | 0.67 | 1.45 | Ref |
| Energy use  (Average monthly MJ/account) | D | 990 | 1194 | 1391 | 1473 | 1696 | 2317 | 0.93 |
|  | C | 906 | 1208 | 1463 | 1468 | 1694 | 2319 | 0.89 |
|  | B | 980 | 1138 | 1310 | 1420 | 1692 | 2289 | 0.48 |
|  | A | 1005 | 1262 | 1457 | 1444 | 1648 | 1819 | Ref |
| SAIFI | D | 0.07 | 0.26 | 0.69 | 0.76 | 1.34 | 2.57 | 0.02 |
|  | C | 0.10 | 0.58 | 0.80 | 0.88 | 1.36 | 2.74 | <0.01 |
|  | B | 0.07 | 0.29 | 0.80 | 0.72 | 1.00 | 1.76 | 0.01 |
|  | A | 0.07 | 0.08 | 0.33 | 0.46 | 0.80 | 1.42 | Ref |

**Supplementary Table S3**. Summary statistics of electricity inaccessibility metrics by HOLC grades.


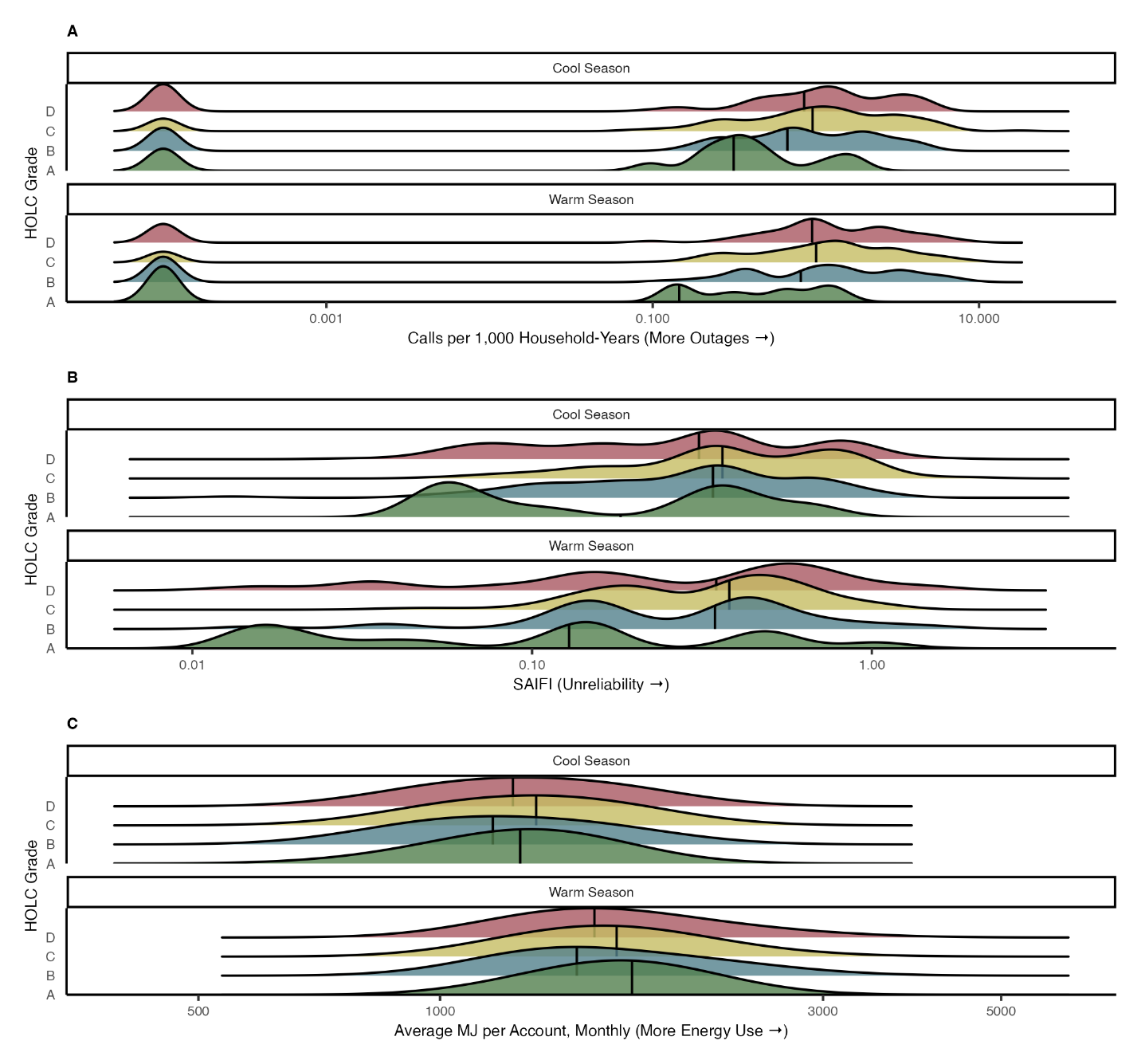


**Supplementary Figure S3**: Prevalence of electricity inaccessibility metrics by cool and warm season for HOLC grades in New York City. Cool season refers to October – April, and warm season refers to May – September.

**
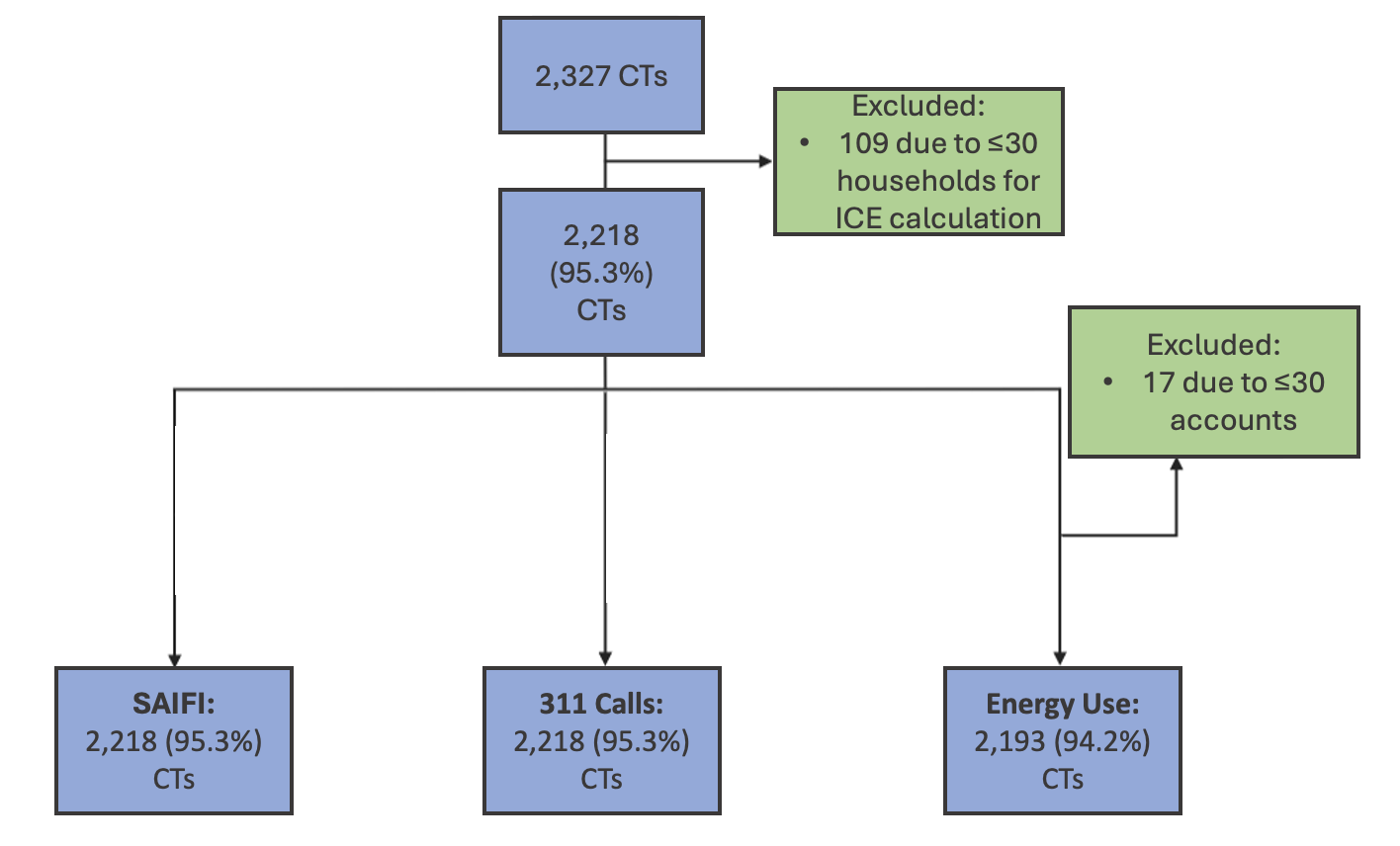
**

**Supplementary Figure S4.** Flowchart of NYC census tracts included in the study after exclusion criteria.

**
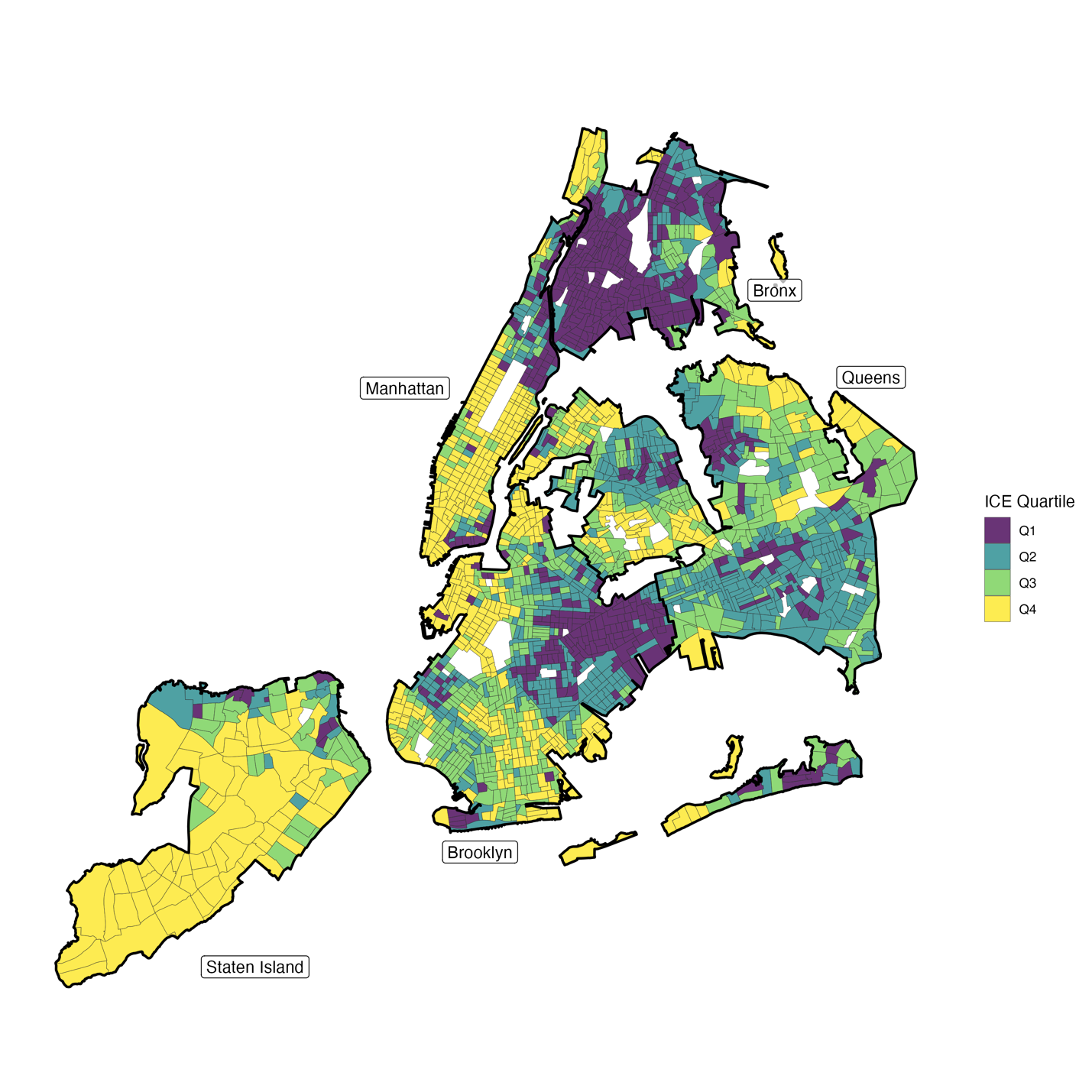
**

**Supplementary Figure S5.** NYC racial and economic ICE quartiles. Q1 is the most disadvantaged, while Q4 is the most advantaged.

|  | ICE quartile | Min. | Q1 | Median | Mean | Q3 | Max. | P-values |
| --- | --- | --- | --- | --- | --- | --- | --- | --- |
| 311 outage rates (calls/1000 customers) | Q1 | 0 | 0.83 | 2.20 | 2.84 | 4.01 | 15.11 | <0.01 |
|  | Q2 | 0 | 0.72 | 1.66 | 2.17 | 2.78 | 16.64 | <0.01 |
|  | Q3 | 0 | 0.28 | 0.79 | 1.24 | 1.49 | 43.73 | <0.01 |
|  | Q4 | 0 | 0 | 0.41 | 0.76 | 0.84 | 47.03 | Ref |
| Energy use  (Average monthly MJ/account) | Q1 | 904 | 1147 | 1234 | 1272 | 1358 | 1958 | <0.01 |
|  | Q2 | 904 | 1150 | 1394 | 1401 | 1621 | 1958 | 0.28 |
|  | Q3 | 886 | 1149 | 1356 | 1374 | 1549 | 1958 | 0.63 |
|  | Q4 | 888 | 1163 | 1325 | 1404 | 1558 | 2318 | Ref |
| SAIFI | Q1 | 0.07 | 0.25 | 0.45 | 0.61 | 0.76 | 2.58 | 0.02 |
|  | Q2 | 0.07 | 0.43 | 0.69 | 0.89 | 1.41 | 2.75 | <0.01 |
|  | Q3 | 0.05 | 0.36 | 0.58 | 0.70 | 0.92 | 2.87 | <0.01 |
|  | Q4 | 0.02 | 0.24 | 0.43 | 0.53 | 0.70 | 2.74 | Ref |

**Supplementary Table S4**. Summary statistics of electricity inaccessibility metrics by present-day census tract ICE quartiles. Q1 is the most disadvantaged, while Q4 is the most advantaged.


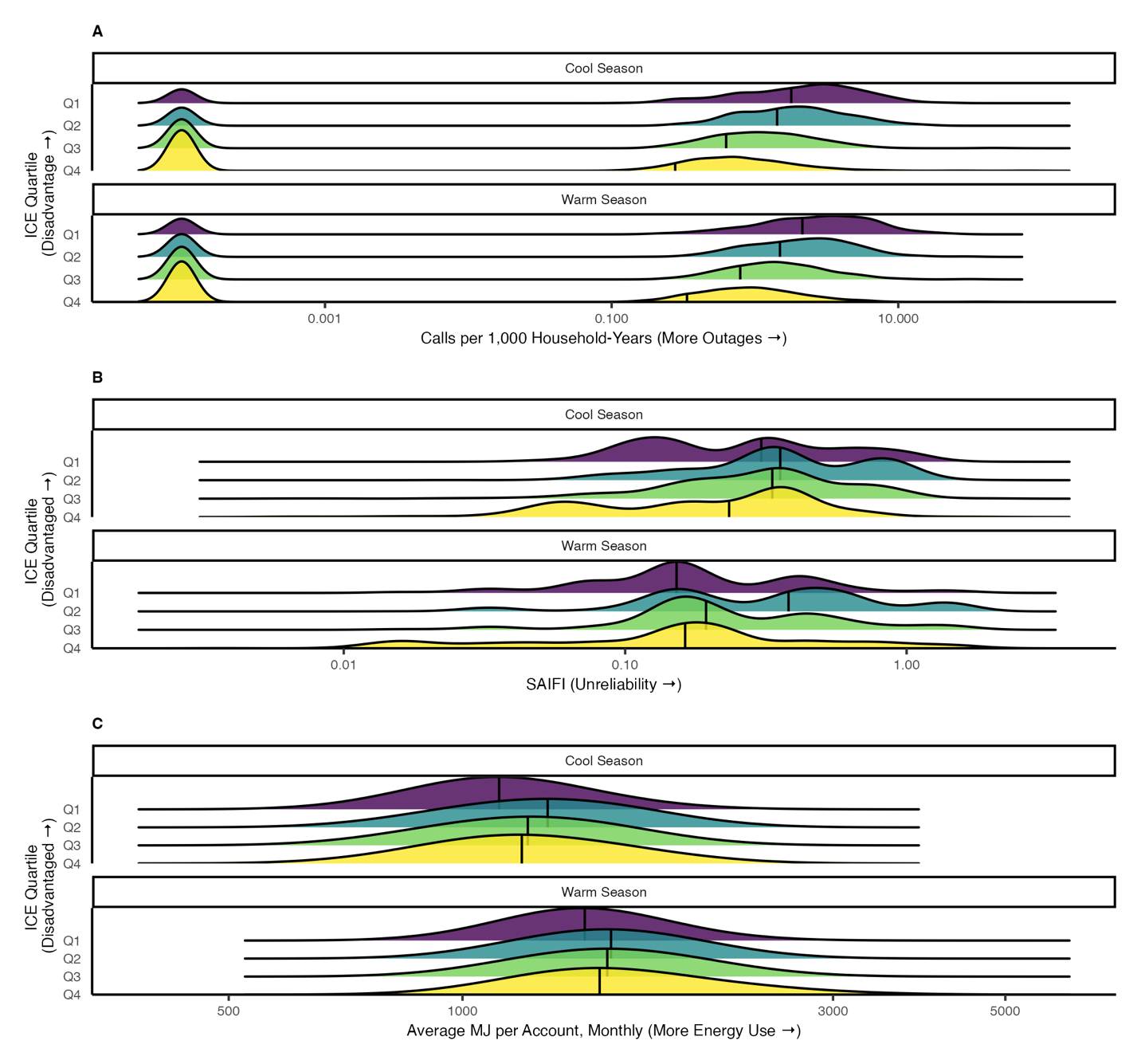


**Supplementary Figure S6**: Prevalence of electricity inaccessibility metrics by cool and warm season for census tract level Index of Concentration quartiles in New York City. Cool season refers to October – April, and warm season refers to May – September.


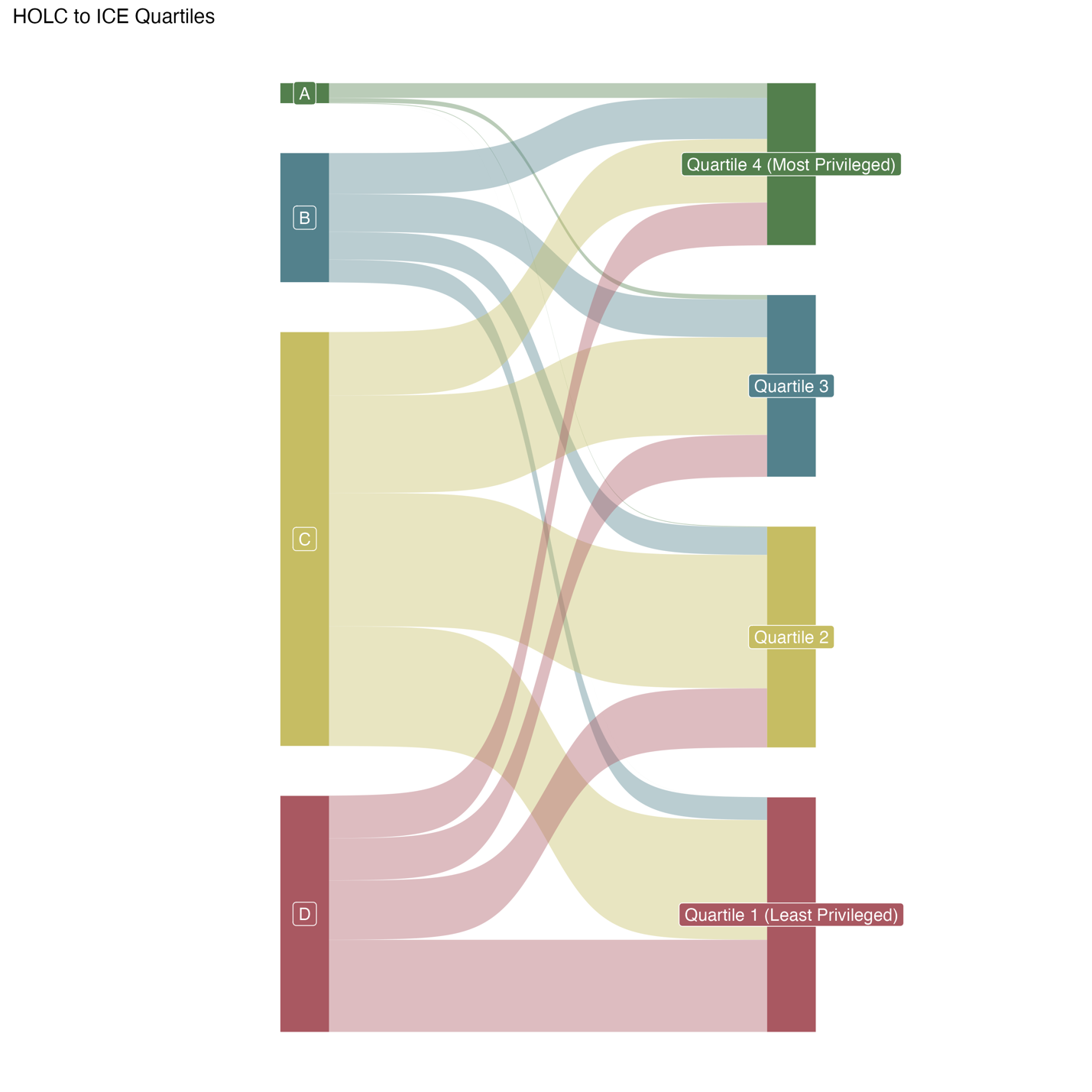


**Supplementary Figure S7**. Longitudinal change in census tracts designated by historical HOLC grade and present-day Index of Concentration at the Extremes. This figure excludes areas without a historical designated HOLC grade.

| HOLC grade | ICE quartile | Number of areas in ICE quartile | Percent |
| --- | --- | --- | --- |
| A (n = 1047) | Q1 (Least Privileged) | 2 | 0.2 |
|  | Q2 | 38 | 3.6 |
|  | Q3 | 265 | 25.3 |
|  | Q4 (Most Privileged) | 742 | 70.9 |
| B (n = 6758) | Q1 (Least Privileged) | 1189 | 17.6 |
|  | Q2 | 1507 | 22.3 |
|  | Q3 | 1764 | 26.1 |
|  | Q4 (Most Privileged) | 2298 | 34.0 |
| C (n = 21665) | Q1 (Least Privileged) | 6117 | 28.2 |
|  | Q2 | 7115 | 32.8 |
|  | Q3 | 5390 | 24.9 |
|  | Q4 (Most Privileged) | 3043 | 14.0 |
| D (n = 12359) | Q1 (Least Privileged) | 4965 | 40.1 |
|  | Q2 | 3128 | 25.3 |
|  | Q3 | 2045 | 16.5 |
|  | Q4 (Most Privileged) | 2229 | 18.0 |
| None (n = 21340) | Q1 (Least Privileged) | 3509 | 16.4 |
|  | Q2 | 4043 | 18.9 |
|  | Q3 | 6308 | 29.5 |
|  | Q4 (Most Privileged) | 7548 | 35.3 |

**Supplementary Table S5**. Prevalence of 0.1 km^2^ areal units by HOLC grade and ICE quartile.

|  | Consistently Advantaged or Persistently Disadvantaged | Min. | Q1 | Median | Mean | Q3 | Max. |
| --- | --- | --- | --- | --- | --- | --- | --- |
| 311 outage rates (calls/1000 customers) | Advantaged | 0 | 0.12 | 0.54 | 0.83 | 1.10 | 5.57 |
|  | Disadvantaged | 0 | 0.71 | 1.82 | 2.47 | 3.42 | 15.2 |
| Energy use  (Average monthly MJ/account) | Advantaged | 978 | 1149 | 1284 | 1347 | 1539 | 2073 |
|  | Disadvantaged | 904 | 1168 | 1317 | 1345 | 1494 | 1958 |
| SAIFI | Advantaged | 0.07 | 0.24 | 0.43 | 0.54 | 0.80 | 2.74 |
|  | Disadvantaged | 0.07 | 0.28 | 0.63 | 0.75 | 1.34 | 2.75 |

**Supplementary Table S6**. Summary statistics of electricity inaccessibility metrics by consistent advantage and persistent disadvantage.
